# Clinical distribution of carbapenem genotypes and resistance to ceftazidime-avibactam in Enterobacteriaceae bacteria

**DOI:** 10.3389/fcimb.2024.1345935

**Published:** 2024-03-20

**Authors:** Xueyun Zhu, Caixia Guo, Shengxi Xu, Fei Lv, Zhusheng Guo, Sisi Lin, CongZhu Yang, Zhuliang Deng, Shaofeng Chen, Ya Huang, Zuguo Zhao, Lu Li

**Affiliations:** ^1^ Guangdong Provincial Key Laboratory of Medical Molecular Diagnostics, School of Medical Technology, The First Dongguan Affiliated Hospital, Guangdong Medical University, Dongguan, China; ^2^ Department of Laboratory Medicine, Dongguan Kanghua Hospital, Dongguan, China; ^3^ The Fourth Affiliated Hospital of Guangzhou Medical University, Guangzhou, China; ^4^ Department of Laboratory Medicine, Dongguan Songshan Lake Tungwah Hospital, Dongguan, China

**Keywords:** Carbapenem-resistant Enterobacteriaceae, BlaNDM, blaKPC, GeneXpert Carba-R, ceftazidime-avibactam

## Abstract

**Introduction:**

Bacterial resistance is a major threat to public health worldwide. To gain an understanding of the clinical infection distribution, drug resistance information, and genotype of CRE in Dongguan, China, as well as the resistance of relevant genotypes to CAZ-AVI, this research aims to improve drug resistance monitoring information in Dongguan and provide a reliable basis for the clinical control and treatment of CRE infection.

**Methods:**

VITEK-2 Compact automatic analyzer was utilized to identify 516 strains of CRE collected from January 2017 to June 2023. To determine drug sensitivity, the K-B method, E-test, and MIC methods were used. From June 2022 to June 2023, 80 CRE strains were selected, and GeneXpert Carba-R was used to detect and identify the genotype of the carbapenemase present in the collected CRE strains. An in-depth analysis was conducted on the CAZ-AVI *in vitro* drug sensitivity activity of various genotypes of CRE, and the results were statistically evaluated using SPSS 23.0 and WHONET 5.6 software.

**Results:**

This study identified 516 CRE strains, with the majority (70.16%) being *K.pneumoniae*, followed by *E.coli* (18.99%). Respiratory specimens had highest detection rate with 53.77% identified, whereas urine specimens had the second highest detection rate with 17.99%. From June 2022 to June 2023, 95% of the strains tested using the CRE GeneXpert Carba-R assay possessed carbapenemase genes, of which 32.5% were *bla_NDM_* strains and 61.25% *bla_KPC_* strains. The results showed that CRE strains containing *bla_KPC_* had a significantly higher rate of resistance to amikacin, cefepime, and aztreonam than those harboring *bla_NDM_*.

**Conclusions:**

The CRE strains isolated from Dongguan region demonstrated a high resistance rate to various antibiotics used in clinical practice but a low resistance rate to tigecycline. These strains produce Class A serine carbapenemases and Class B metals β-lactamases, with the majority of them carrying *bla_NDM_* and *bla_KPC_*. Notably, CRE strains with *bla_KPC_* and *bla_NDM_* had significantly lower resistance rates to tigecycline. CAZ-AVI showed a good sensitivity rate with no resistance to CRE strains carrying *bla_KPC_*. Therefore, CAZ-AVI and tigecycline should be used as a guide for rational use of antibiotics in clinical practice to effectively treat CRE.

## 1 Introduction

Bacterial resistance is a major threat to public health worldwide. According to the latest data released by the 2021 National Bacterial Resistance Monitoring Network (CARSS) in 2023, 71.1% of clinical pathogens will be gram-negative bacteria (China Antimicrobial Resistance Surveillance System (CARSS), 2023b). Among them, intestinal bacteria are the most common drug-resistant bacteria in clinical practice, and carbapenem antibiotics include meropenem, imipenem, and ertapenem, it is one of the most effective antibiotics for treating infections caused by multidrug-resistant *Enterobacteriaceae* (MDR-E) (Djukovic et al., 2022; Esemu et al., 2022; Zheng et al., 2022; Lyu et al., 2023). With the widespread application of carbapenems in clinical treatment, the detection rate of carbapenem-resistant *Enterobacteria (*CR-Ent) has rapidly increased. The results revealed that the most common human-source CR-Ent species in China was *E. xiangfangensis* (66/92, 71.93%), and the proportion of carbapenemase-producing CP-Ent in CR-Ent was higher (72/92, 78.26%) than that in other global regions (Zhu et al., 2023). It mainly causes lower respiratory tract infections (65.4%), urinary tract infections (16.6%), abdominal infections (7.7%), and bacteremia (7.7%), with a total hospital mortality rate of 33.5% (Zhang et al., 2018). Currently, over 20% of *Enterobacteriaceae* have developed resistance, and it has been found that the production of carbapenemases is the main mechanism of resistance to carbapenems in *Enterobacteriaceae* bacteria (Tzouvelekis et al., 2012; Pang et al., 2018; Wang et al., 2018; Hu et al., 2019).

Ceftazidime-avibactam (CAZ-AVI) was awarded the Qualified New Antibiotic Qualification (QIDP), approved in the United States in 2015 and the European Union in 2016, and is now available in more than 40 countries and regions worldwide. Approved by the National Drug Administration (CFDA) on May 21, 2019, for the treatment of complex intraperitoneal infections (cIAI), hospital-acquired pneumonia (HAP)/ventilator-associated pneumonia (VAP), caused by *Klebsiella pneumoniae (K. pneumoniae), Enterobacter cloacae, Escherichia coli (E. coli), Proteus mirabilis and Pseudomonas aeruginosa (P. aeruginosa)*, aged ≥18 years. CAZ-AVI can combat infections caused by most MDR-E strains, including carbapenem-resistant *Enterobacteriaceae* (CRE) (Zhen and Feng, 2021). CAZ-AVI) is composed of the third-generation cephalosporin ceftazidime with the novel non-β-lactamase β-lactamase inhibitor abvibatam (AVI), which inhibits class A, C, and some class D enzymes. The essential difference between AVI and classical β-lactamase lies in the fact that the serine of β-lactamase binds to the AVI amide bond to form a covalent conjugate to obtain the enzyme inhibitor complex. This enzyme-inhibiting form does not hydrolyze AVI, and AVI can recover its activity after cycling to form a lactam ring (Sader et al., 2015; Wong and van Duin, 2017). During this process, the structure of AVI is restored through a reversible reaction, resulting in a long-term inhibitory effect on enzymes. Moreover, AVI does not induce β-lactamase production (Ehmann et al., 2013; Livermore et al., 2015), and it was also found that 1-5 AVl molecules can inhibit one β-lactamase molecules, while 55 to 214 molecules are required for trizobactam and clavulanic acid, therefore AVI has stronger antibacterial effects (Zhanel et al., 2013). In addition, clinical experimental results have shown that patients infected with CRE strains treated with CAZ-AVI after ineffective treatment with other antibiotics had a cure rate of 95% (Temkin et al., 2017). This indicates that CAZ-AVI has a strong antibacterial effect on CRE bacteria and is an effective drug for the clinical treatment of infected CRE strains. However, there is no universally effective method for rapid identification of CRE strains in clinical practice.

Therefore, this study aimed to use the GeneXpert Carba-R method to quickly and accurately identify the carbapenase genotypes of *Enterobacteriaceae.* The study also investigated the *in vitro* antibacterial activity of CAZ-AVI against different carbapenase genotypes of CRE strains through an antibiotic susceptibility test (AST), providing a new strategy and a theoretical basis for precise drug use in patients with clinical infection.

## 2 Experimental materials and methods

### 2.1 Experimental strains

From January 2017 to June 2023, 516 Dongguan were collected from Dongguan, and duplicate samples were eliminated. The quality control strain is *Escherichia coli* ATCC 25922. CRE strains were mainly derived from respiratory, urine, blood, ascites, pus, and other specimens.

### 2.2 Identification of bacterial strains and AST

Strain identification and AST were performed according to the procedures recommended in the National Clinical Testing Procedures of the VITEK-2 Compact Automated Bacterial Identification and Drug Sensitivity System Analyzer (VITEK-2 Compact automatic analyzer) (Merieux, France). AST results were strictly determined according to the standards of the Clinical Laboratory Standardization Institute (CLSI) of the United States. The disc diffusion test (K-B method) and culture medium were purchased from OXOID (UK). Tigecycline was tested for minimum inhibitory concentration (MIC) methods using an E-test strip (BioMerier, France), and the results were determined in accordance with the guidelines of the United States Food and Drug Administration (FDA).

### 2.3 Detection of carbapenemase gene

The GeneXpert Carba-R detection method was adopted, and the specific steps were as follows: First of all, the bacterial solution was adjusted to a turbidity of 0.5 MCG with normal saline, 10 μL bacterial solution was absorbed into the sample processing solution, and then oscillated with an oscillator for 10s. Finally, 1.7 mL of the mixed solution was added to the Carba-R reagent kit, and the Cepheid^®^ GeneXpert^®^ Infinity System Fully Automated Medical PCR Analysis System (Infinity-80) (Cepheid (Shanghai) Trading Co., LTD.) was used for detection.

### 2.4 Statistical processing

WHONET 5.6 software was used for the statistical analysis of strain distribution and AST. *SPSS* software (version 23.0) and Fisher’s exact probability test were used to statistically analyze the differences in drug resistance rates of CRE strains, and *P*<0.05 indicated that the differences were statistically significant.

## 3 Results

### 3.1 Specimen sources of CRE strains

**Figure 1:**
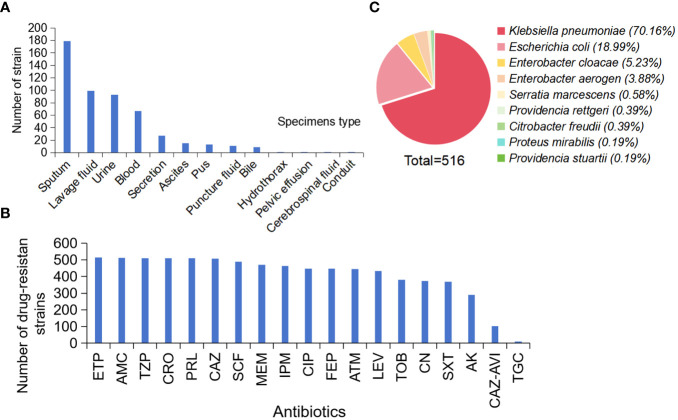
Distribution and AST Results of CRE Strains. **(A)** Sample distribution, **(B)** AST Results, **(C)** Distribution of 516 strains. (Ertapenem, ETP; Piperacillin-tazobactam, TZP; Ceftriaxone, CRO; Piperacillin, PRL; Ceftazidime, CAZ; Cefoperazone-sulbactam, SCF; Meropenem, MEM; Imipenem, IPM; Ciprofloxacin, CIP; Cefepime, FEP; Aztreonam, ATM; Levofloxacin, LEV; Tobramycin, TOB; Gentamicin, CN; Sulfamethoxazole-trimethoprim, SXT; Amikacin, AK; Tigecycline, TGC).

CRE strains were mainly isolated from respiratory (278 strains, 53.77%), urine (93 strains, 17.99%), blood (67 strains, 12.96%), ascites (15 strains, 2.90%), pus (13 strains, 2.51%), and other specimens (24 strains, 4.63%) (**Figure 1A**).

In addition, 49 strains of *bla_KPC_* were mainly distributed in the purum (28 strains, 57.1%), lavage fluid (seven strains, 14.3%), urine (five strains, 10.2%), blood (five strains, 10.2%), functional fluid (two strains, 4.1%), wound secrets (one strain, 2%), and bile (one strain, 2%). 27 strains of *bla_NDM_* were mainly distributed in the purum (nine strains, 33.3%), wound secrets (seven strains, 25.9%), urine (four strains, 14.8%), blood (three strains, 11.1%), bile (one strain, 3.7%), ascites (one strain, 3.7%), pelvic fusion (one strain, 3.7%), and drainage fluid (one strain, 3.7%) (**Table 1**).

**Table 1:** Proportion of *bla_KPC_*, *bla_NDM_* genes in various specimen types.

Specimens type	*bla*_KPC_ (Number)	*bla*_NDM_ (Number)	No genotype detected (Number)
Sputum	28	9	1
Lavage fluid	7	0	0
Urine	5	4	2
Blood	5	3	0
Puncture fluid	2	0	0
Wound secretion	1	7	0
Bile	1	1	0
Ascites	0	1	0
Pelvic effusion	0	1	0
Drainage fluid	0	1	1

### 3.2 Resistance of CRE strains to commonly used antibiotics

The AST assay of the 516 CRE strains showed that the drug resistance rate to ertapenem was the highest (99.6%). The drug resistance rate to piperacillin-tazobactam, ceftriaxone, and piperacillin was 98.8%. In addition, the drug resistance rates for ceftazidime, cefoperazone-sulbactam, meropenem, and imipenem were > 90% (98.3%, 95%, 91.3%, and 90.1%, respectively). Tigecycline resistance was the lowest (2.1%) (**Figure 1B**).

### 3.3 Sample distribution of CRE

Analysis of 516 CRE strains collected showed that 362 strains of *K. pneumoniae* (Vading et al., 2011; Shen et al., 2022) (70.16%), 98 strains of *E. coli* (18.99%), 27 strains of *Enterobacter cloacae* (5.23%), 20 strains of *Enterobacter aerogenes* and 3 other strains (3.88%), 3 strains of *Serratia marcescens* (0.58%). There were two strains of *Providencia stuartii* (0.39%), two strains of *Proteus mirabilis* (0.39%), one strain of *Provencia steinii* (0.19%), and one strain of *Proteus mirabilis* (0.19%) (**Figure 1C**).

### 3.4 Carbapenemase gene was detected by GeneXpert Carba-R assay

The GeneXpert Carba-R assay was used to detect 80 strains collected between June 2022 and June 2023. It was found that 76 strains (95%) carried carbapenase gene, among which 27 strains of *bla_NDM_* (32.5%) and 49 strains of *bla_KPC_* (61.25%) carried carbapenase gene. *bla_VIM_*, *bla_IPM_* and *bla_OXA_* genes were not detected, and no strains containing both *bla_NDM_* and *bla_KPC_* were detected (**Table 2**).

**Table 2:** The distribution of carbapenemase genotypes of CRE strains.

Strains	*bla*_KPC_ (Number)	*bla*_NDM_ (Number)	No genotype detected (Number)
*Klebsiella pneumoniae*	48	4	2
*Escherichia coli*	1	18	1
*Serratia marcescens*	0	1	0
*Enterobacter cloacae*	0	3	0
*Providencia stuartii*	0	1	0
*Enterobacter aerogen*	0	0	1

### 3.5 AST results of CRE strains with different carbapenemase genotypes

Because of the different clinical treatment regimens used for patients infected with different carbapenem enzyme types, we tested the AST of CRE strains carrying *bla_KPC_* and *bla_NDM_*. The results showed that the resistance rate of CRE strains to commonly used antibiotics was generally high, with CRE strains carrying the *bla_KPC_* genotype having a lower resistance rate to tigecycline (13.9%), The resistance rate of CRE strains carrying the *bla_NDM_* genotype to amikacin (22.22%) and tigecycline (7.14%) is relatively low. According to Fisher’s exact probability test, the resistance rate of CRE strains carrying the *bla_KPC_* genotype to cefepime, amikacin, and aztreonam was significantly higher than that of strains carrying the *bla_NDM_* genotype (P<0.05), whereas there was no significant difference between them in resistance rates to other antibiotics (P>0.05). In addition, we found that none of the CRE strains carrying the *bla_KPC_* genotype showed resistance to ceftazidime-avibactam, while the *bla_NDM_* genotype showed 100% resistance to ceftazidime-avibactam (**Table 3**).

**Table 3:** Antibiotic susceptibility test results of CRE strains carrying *bla*_KPC_ and *bla*_NDM_.

Antibiotics	*bla*_KPC_ (n=49)		*bla*_NDM_ (n=27)		*P*
	drug resistance rate (%)	Number	drug resistance rate (%)	Number	
Amoxicillin-clavulanate	100	49	100	27	–
Piperacillin-tazobactam	100	49	100	27	–
Ceftazidime-avibactam	0	0	100	27	–
Ceftazidime	100	49	100	27	–
Ceftriaxone	100	49	100	27	–
Ertapenem	100	49	100	27	–
Cefepime	100	49	77.77	21	0.001354
Imipenem	93.88	46	100	27	0.548
Aztreonam	93.88	46	62.96	17	0.000614
Levofloxacin	87.76	43	85.18	23	0.751
Amikacin	67.35	33	22.22	6	0.000165
Tigecycline	12.24	6	7.41	2	0.511

“-” indicates that the P value cannot be calculated.

## 4 Discussion

According to the Ambler molecular classification method, carbapenemases can be classified into categories A, B, and D. Among the 4 classes of β-lactamases defined by the Ambler classification system, the carbapenemas that confer carbapenem resistance in Enterobacteriaceae belong to 3 of them: Class A (*K. pneumoniae* carbapenemas, KPC), Class B (metallo-β-lactamases, MBL including New Delhi metallo-β-lactamases, NDM) and Class D (OXA-48 like carbapenemases) (van Duin and Doi, 2017). Class A is serine carbapenemase, mainly consisting of *bla*_KPC_ (*bla*_KPC-2_-*bla*_KPC-55_)、*bla*_SME_ (*bla*_SME-1_-*bla*_SME-5_)、*bla*_IMI_ (*bla*_IMI-1_-*bla*_IMI-18_)、*bla*_NMC_ and *bla*_GES_ (*bla*_GES-1_-*bla*_GES-43_); Class B is metallo-β-lactamases, mainly *bla*_NDM_ (*bla*_NDM-1_-*bla*_NDM-29_)、*bla*_IMP_ (*bla*_IMP-1_-*bla*_IMP-85_)、*bla*_VIM_ (*bla*_VIM-1_-*bla*_VIM-69_)、*bla*_GIM_ (*bla*_GIM-1_-*bla*_GIM-2_), and *bla*_SPM_; Class D is OXA-48-like carbapenemases, mainly *bla*_OXA-181_ and *bla*_OXA-232_ (Yu et al., 2020, Yu et al., 2022). In addition to producing carbapenemases, the mechanism of carbapenem resistance in some strains is the production of ultra-broad spectrum β-lactam enzyme and/or AmpC enzyme combined with downregulation or deletion of outer membrane porin expression (Zhang et al., 2017; Fan et al., 2023).

The carbapenemases produced by CRE strains clinically isolated in China are mainly of the KPC and NDM types, with some strains producing OXA-48, IMP, and VIM carbapenemases (Zhang et al., 2017; Wang et al., 2018; Han et al., 2020). The main subtype of KPC-type carbapenemases is KPC-2, the main subtypes of NDM-type metalloenzymes are NDM-1 and NDM-5, and the main subtypes of OXA-48 type carbapenemases are OXA-181 and OXA-232 enzyme types (Yu et al., 2020, Yu et al., 2022). The CHINET surveillance of antimicrobial resistance among bacterial isolates from 2005 to 2022 showed that the resistance rate of *K. pneumoniae* strains isolated clinically in China to carbapenem antibiotics increased from 3% in 2005 to over 24.2% in 2022, an 8-fold increase (Hu et al., 2020; Zheng et al., 2020). According to data from the China Antimicrobial Resistance Surveillance System (CARSS) in 2018, the average resistance rate of *K. pneumoniae* clinically isolated from 1429 hospitals nationwide to carbapenems is 10.1% and exceeded 20% in some provinces and cities (China Antimicrobial Resistance Surveillance Network, 2020). The detection rate of carbapenem-resistant *K. pneumoniae* (CR-KPN) in 2021 is 11.3%, an increase of 0.4% compared to 10.9% in 2020 (China Antimicrobial Resistance Surveillance Network, 2021; China Antimicrobial Resistance Surveillance System (CARSS), 2023a). This indicates that the resistance rate of *K. pneumoniae* to carbapenems is gradually increasing and the situation is severe. However, there are certain differences in the carbapenem-producing enzyme types of different bacteria in different regions, which lead to different clinical treatment plans for different carbapenem-producing enzyme types. Therefore, there is an urgent need to develop new methods for the rapid and accurate identification of carbapenem-producing Enterobacteriaceae enzymes and test their drug sensitivity results to guide the clinical adoption of correct treatment plans, effectively treat patients’ infections, and save treatment time.

According to data from the International Network for Optimal Resistance Monitoring (INFORM) data, the resistance rate of meropenem-resistant *E. coli* to CAZ-AVI was 27% between 2015 and 2017 (Spiliopoulou et al., 2020). According to INFORM reports, the resistance rate of most Enterobacteriaceae bacteria to CAZ-AVI is low (<2.6%) (Wise et al., 2018), whereas the resistance rate of *P. aeruginosa* is relatively high, reaching 4%–8% (Nichols et al., 2016). Several studies conducted from 2006 to 2018 have shown that the resistance rates of most gram-negative bacteria to CAZ-AVI were below 3.7% in the United States (Senchyna et al., 2019). In Europe and the Asia-Pacific region, the resistance rates of Enterobacteriaceae to CAZ-AVI are less than 1.1% and 1.7%, respectively, and those of *P. aeruginosa* are less than 8.9% and 7.4%, respectively (Karlowsky et al., 2018; Kazmierczak et al., 2018). In Canada and Brazil, the rate of resistance to CAZ-AVI is generally below 5.3% (Denisuik et al., 2015; Rossi et al., 2017). Therefore, CAZ-AVI could be considered as an adequate treatment option for tract infections caused by KPC and OXA-48 producers (García-Castillo et al., 2018).

The results of this study showed that *K. pneumoniae* was the main CRE strain isolated from the Dongguan area, primarily from respiratory specimens. The AST assay showed that CRE strains isolated from Dongguan had the lowest tigacycline resistance. In addition, 76 strains (92.5%) carrying the carbapenemase gene were detected using the GeneXpert Carba-R method, among which 27 strains contained *bla*_NDM_ gene (33.75%) and 24 strains contained *bla*_KPC_ gene (61.25%). No *bla*_VIM_, *bla*_IPM_, or *bla*_OXA_ genes were detected and no strains with both *bla*_NDM_ and *bla*_KPC_ were detected. It is speculated that CRE strains may be caused by factors such as high yield of AMPCase, ultra-broad spectrum β-lactamase, deletion of outer membrane protein, and overexpression of efflux pump (Li et al., 2012). Therefore, the results of this study indicate that CRE strains in Dongguan region were mainly serine-producing carbapenemase and B-producing metal β-lactamase.

Current studies have shown that the resistance mechanisms of CAZ-AVI are mainly the following: (1) expression of metallic β-lactamase (Lahiri et al., 2015; Aitken et al., 2016; Grupper et al., 2017); (2) promote the expression of *bla*_KPC_ gene and the mutation of key sites of β-lactamase (Giddins et al., 2017; Gaibani et al., 2018); (3) porin deletion changes membrane permeability (Winkler et al., 2015; Humphries and Hemarajata, 2017; Nelson et al., 2017; Rocker et al., 2020; Guo et al., 2021); (4) promote the expression of efflux pumps (Zhang et al., 2017). One of the most common resistance mechanisms is the production of metallic β-lactamase. Class B metallic β-lactamases bind to β-lactamides substrates via zinc ions to hydrolyze all clinically used serine β-lactamase inhibitors, including avibactam (Schillaci et al., 2017), suggesting that CAZ-AVI cannot be used to treat patients infected with strains producing such enzymes. Mutations in the KPC-type carbapenemase gene are the main mechanism leading to CAZ-AVI resistance. Moreover, studies have shown that β-lactamase amino acid mutations or deletion, membrane permeability defects (i.e., changes in OmpK35, OmpK36, and OmpK37), and penicillin-binding protein mutations, and overexpression of KPC and ESBL determinants (SHV-, CTX-M-, or VEB types) are all associated with the resistance of KPC type carbapenemase strain to CAZ-AVI. In this study, we found significant differences in the resistance rates of CRE strains carrying *bla*_KPC_ and *bla*_NDM_ to cefepime, aztreonam, and amikacin, and no strains carrying *bla*_KPC_ were found to be resistant to CAZ-AVI, suggesting a reason for the low frequency of CAZ-AVI antibiotic use in Dongguan. This study found that CRE strains carrying the *bla*_NDM_ gene were all resistant to CAZ-AVI. In summary, the GeneXpert Carba-R method can rapidly detect the genotype of carbapenemase carried by CRE strains, save time for the treatment of patients with clinical emergency infections, and provide an experimental basis for the clinical use of CAZ-AVI for the treatment of infections. It is important to provide targeted and personalized treatment in clinical departments.

Finally, our study had some limitations. First, because of the large sample size of the carbapenem-resistant Enterobacteriaceae strains collected and the lack of research funding, we only used GeneXpert Carba-R to detect and identify the genotype of carbapenemase in CRE strains from 2022 to 2023. Therefore, we did not analyze all strains from 2017 to 2023. Second, this single-center study was conducted at a comprehensive tertiary hospital in Dongguan. The sample size of CRE strains was relatively small; therefore, our results cannot be extrapolated to other hospitals and regions in China. Additionally, this study lacks relevant research on the mechanisms of drug resistance is lacking. In future studies, we will conduct relevant analyses of CRE strain resistance mechanisms to gain a more comprehensive understanding of CRE strain resistance in the Dongguan region.

## 5 Conclusion

In summary, the CRE strains isolated from the Dongguan region demonstrated a high resistance rate to various antibiotics used in clinical practice but a low resistance rate to tigecycline. These strains produce Class A serine carbapenemases and Class B metals β-lactamases, with the majority of them carrying *bla*_NDM_ and *bla*_KPC_. Notably, CRE strains with *bla*_KPC_ and *bla*_NDM_ had significantly lower resistance rates to tigecycline. CAZ-AVI showed a good sensitivity rate with no resistance to CRE strains carrying *bla*_KPC_. However, the CRE strains with *bla*_NDM_ were not sensitive to CAZ-AVI. Therefore, CAZ-AVI and tigecycline should be used as a guide for the rational use of antibiotics in clinical practice in to effectively treat CRE. There is a need to conduct future analyses of CRE strain resistance mechanisms to gain a more comprehensive understanding of CRE strain resistance.

## Data availability statement

The original contributions presented in the study are included in the article/supplementary material. Further inquiries can be directed to the corresponding authors.

## Author contributions

XZ: Data curation, Formal analysis, Writing – original draft. CG: Data curation, Supervision, Visualization, Writing – original draft. SX: Investigation, Methodology, Writing – original draft. FL: Software, Writing – original draft. ZG: Project administration, Writing – original draft. SL: Data curation, Writing – original draft. CY: Investigation, Writing – original draft. ZD: Methodology, Software, Writing – original draft. SC: Conceptualization, Writing – original draft. YH: Writing – original draft. ZZ: Supervision, Writing – original draft. LL: Funding acquisition, Writing – original draft, Writing – review & editing.
